# External versus internal fixation for bicondylar tibial plateau fractures: systematic review and meta-analysis

**DOI:** 10.1007/s10195-015-0372-9

**Published:** 2015-08-26

**Authors:** David Metcalfe, Craig J. Hickson, Lesley McKee, Xavier L. Griffin

**Affiliations:** Harvard Medical School, 25 Shattuck Street, Boston, MA 02115 USA; Warwick Medical School, Gibbet Hill Road, Coventry, CV4 7AL UK; Division of Trauma, Burns, and Surgical Critical Care, Brigham and Women’s Hospital, 75 Francis Street, Boston, MA 02115 USA; Leicester Royal Infirmary, Infirmary Square, Leicester, LE1 5WW UK; Forth Valley Hospital, Stirling Road, Larbert, Scotland, UK

**Keywords:** External fixation, Internal fixation, Bicondylar tibial plateau, Proximal tibial fracture

## Abstract

**Background:**

It is uncertain whether external fixation or open reduction internal fixation (ORIF) is optimal for patients with bicondylar tibial plateau fractures.

**Materials and methods:**

A systematic review using Ovid MEDLINE, Embase Classic, Embase, AMED, the Cochrane Library, Open Grey, Orthopaedic Proceedings, WHO International Clinical Trials Registry Platform, Current Controlled Trials, US National Institute for Health Trials Registry, and the Cochrane Central Register of Controlled Trials. The search was conducted on 3rd October 2014 and no language limits were applied. Inclusion criteria were all clinical study designs comparing external fixation with open reduction internal fixation of bicondylar tibial plateau fractures. Studies of only one treatment modality were excluded, as were those that included unicondylar tibial plateau fractures. Treatment effects from studies reporting dichotomous outcomes were summarised using odds ratios. Continuous outcomes were converted to standardized mean differences to assess the treatment effect, and inverse variance methods used to combine data. A fixed effect model was used for meta-analyses.

**Results:**

Patients undergoing external fixation were more likely to have returned to preinjury activities by six and twelve months (*P* = 0.030) but not at 24 months follow-up. However, external fixation was complicated by a greater number of infections (OR 2.59, 95 % CI 1.25–5.36, *P* = 0.01). There were no statistically significant differences in the rates of deep infection, venous thromboembolism, compartment syndrome, or need for re-operation between the two groups.

**Conclusion:**

Although external fixation and ORIF are associated with different complication profiles, both are acceptable strategies for managing bicondylar tibial plateau fractures.

**Level of evidence:**

II.

## Introduction

Tibial plateau fractures are uncommon injuries, representing only 1.2 % of all fractures [1]. They have a bimodal incidence, occurring in young patients suffering high-energy trauma, and as fragility fractures in the elderly [2]. Bicondylar tibial plateau fractures (Schatzker types V and VI/Orthopaedic Trauma Association types C1, C2, and C3) typically follow high-energy trauma [3, 4]. They are complex intra-articular injuries with implications for articular congruity, cartilage integrity and extra-articular structures [5]. Associated complications include compartment syndrome, soft tissue damage, secondary osteoarthrosis (OA), and persistent knee instability. Conservative treatment is rarely appropriate for these injuries [6].

Management aims are anatomic reduction of the articular surface, restoration of axial alignment, and stable fixation to prevent secondary displacement of the fracture fragments [7]. A commonly employed technique is open reduction and internal fixation (ORIF), using a plate and screws through either an extended anterior incision or through multiple smaller incisions to preserve the soft tissue envelope. High-energy bicondylar fractures are often already accompanied by soft tissue damage, and ORIF in this setting is associated with wound complications, e.g., skin necrosis and infection [8]. Soft tissue considerations may also delay operative fixation and/or contraindicate ORIF altogether. In addition, there is evidence to suggest that, once alignment is restored, residual articular incongruity may not impair long-term functional results following these injuries [9–13].

These observations have driven a search for alternative interventions, including isolated tension band wire fixation [14], minimally invasive plate osteosynthesis (MIPO) [2], and hybrid external fixation [15]. The latter technique involves reduction of the fracture using closed manipulation, percutaneously, or through limited incisions. Fracture reduction is stabilized with one or more percutaneous lag screws, and an external fixator (typically a circular frame) is assembled to secure the metaphysis to the tibial diaphysis.

This systematic review sought to compare all forms of external fixation (including hybrid techniques) with ORIF for bicondylar tibial plateau fractures in terms of radiological and clinical outcomes as well as their post-operative complication profiles.

## Materials and methods

A systematic review was performed in line with the Cochrane Handbook for Systematic Reviews of Interventions [16] and reported according to the Preferred Reporting Items for Systematic Reviews and Meta-Analysis (PRISMA) statement [17].

### Search strategy

The following databases were searched using the strategy below: Ovid MEDLINE (1946 to September week 4 2014), Embase Classic (1947–1973), Embase (1974 to 2nd October 2014), and AMED (1985 to September 2014). All searches were conducted on 3rd October 2014. No limits were applied in terms of language, publication status, or study design. The search strategy was:

1. “proximal tib*” or “tibial plateau”
2. “schatzker 6” or “schatzker VI” or “schatzker type 6” or “schatzker type VI” or “schatzker 5” or “schatzker V” or “schatzker type 5” or “schatzker type V” or “bicondylar” or “comminuted” or “complex”
3. “complex tibial plateau”
4. “external fix*” or “frame”
5. 1 and 2
6. 3 or 5
7. 4 and 6.

The Cochrane Library and Open Grey (System for Information on Grey Literature in Europe, http://www.opengrey.eu) were searched using the term “tibial plateau”.

Conference proceedings from the British Orthopaedic Association, British Trauma Society, Orthopaedic Trauma Association, British Association for Surgery of the Knee, and European Federation of National Associations for Orthopaedics and Traumatology were screened using the digital archive Orthopaedic Proceedings [18] from 1st March 2002 to 3rd October 2014. Titles and abstracts were searched using the term “tibial plateau fracture”.

Ongoing and recently completed trials were searched using the term “tibial plateau” in the WHO International Clinical Trials Registry Platform [19], Current Controlled Trials [20], US National Institute of Health Trials Registry [21], and the Cochrane Central Register of Controlled Trials [22].

Authors of leading studies were contacted for details of ongoing work. Reviews, editorials, and opinion articles were used as potential sources of further references.

### Inclusion and exclusion criteria

All clinical study designs were included that met the following criteria:

- Reporting on human patients with bicondylar (OTA C1, C2, and C3) tibial plateau fractures.
- Direct comparison between any form of external fixation (including hybrid techniques utilizing percutaneous screw fixation) and ORIF.
- Reporting outcomes that were radiological (fracture reduction, union, subsequent OA) or clinical (functional scores, patient-reported outcomes, need for subsequent operation including arthroplasty), and/or post-operative complications (defined as any deleterious event described by study authors as post-operative complications).

Criteria for excluding studies were:

- Reporting data from patients with peri-prosthetic and/or pathological fractures.
- Failure to analyze data on bicondylar fractures separately, e.g., populations including patients with unicondylar fractures. Authors were contacted for unpublished data in all such cases.
- Isolated case series of patients undergoing either ORIF or external fixation without distinction between treatment modalities.

### Selection of studies

Two authors (DM and CH) independently screened all retrieved items by title then abstract and full text as necessary using the pre-determined selection criteria. Disagreements were resolved through discussion.

### Quality assessment

Two authors (DM and LM) independently assessed risk of bias. Randomized controlled trials were assessed using the Cochrane Collaboration Risk of Bias Tool [16], which considers selection bias (random allocation and allocation concealment), performance bias (blinding of participants and personnel), detection bias (blinding of outcome assessment), attrition bias (incomplete outcome data), reporting bias (selective reporting), and other sources of bias. Non-randomized studies were assessed using the Risk of Bias Assessment Tool for Non-Randomized Studies (RoBANS) [23]. This tool considers similar bias domains to that produced by Cochrane but is modified for non-randomized study designs. Both tools assess risk of bias in each domain as “high”, “low”, or “unknown”. Disagreements were resolved through discussion.

### Extraction of data

A single author (DM) extracted data from studies onto a standardized proforma. Study authors were contacted for clarification and/or additional data when fields could not be completed from the published reports.

### Statistical analysis

Treatment effects from studies reporting dichotomous outcomes were summarised using odds ratios and combined using the Mantel–Haenszel technique [24]. Continuous outcomes were converted to standardized mean differences to assess the treatment effect, and inverse variance methods were used to combine data. Confidence intervals were reported at the 95 % level and a fixed effect model was used for meta-analyses, although we planned to use a random effects model in the event of significant heterogeneity. Statistical heterogeneity was assessed by visual inspection of overlapping confidence intervals on forest plots and consideration of the *I*^2^ with *P* < 0.1 interpreted as significant heterogeneity.

Except for assessment of heterogeneity, *P* < 0.05 was used as the threshold for statistical significance. All statistical analyses were performed using Stata v.13.1 (StataCorp, Memphis, TN) or RevMan v.5.2.3 (Nordic Cochrane Centre, Copenhagen, Denmark). RevMan was also used to construct forest plots.

Missing data that could not be retrieved despite contacting study authors was excluded from the analysis.

## Results

**Fig. 1:**
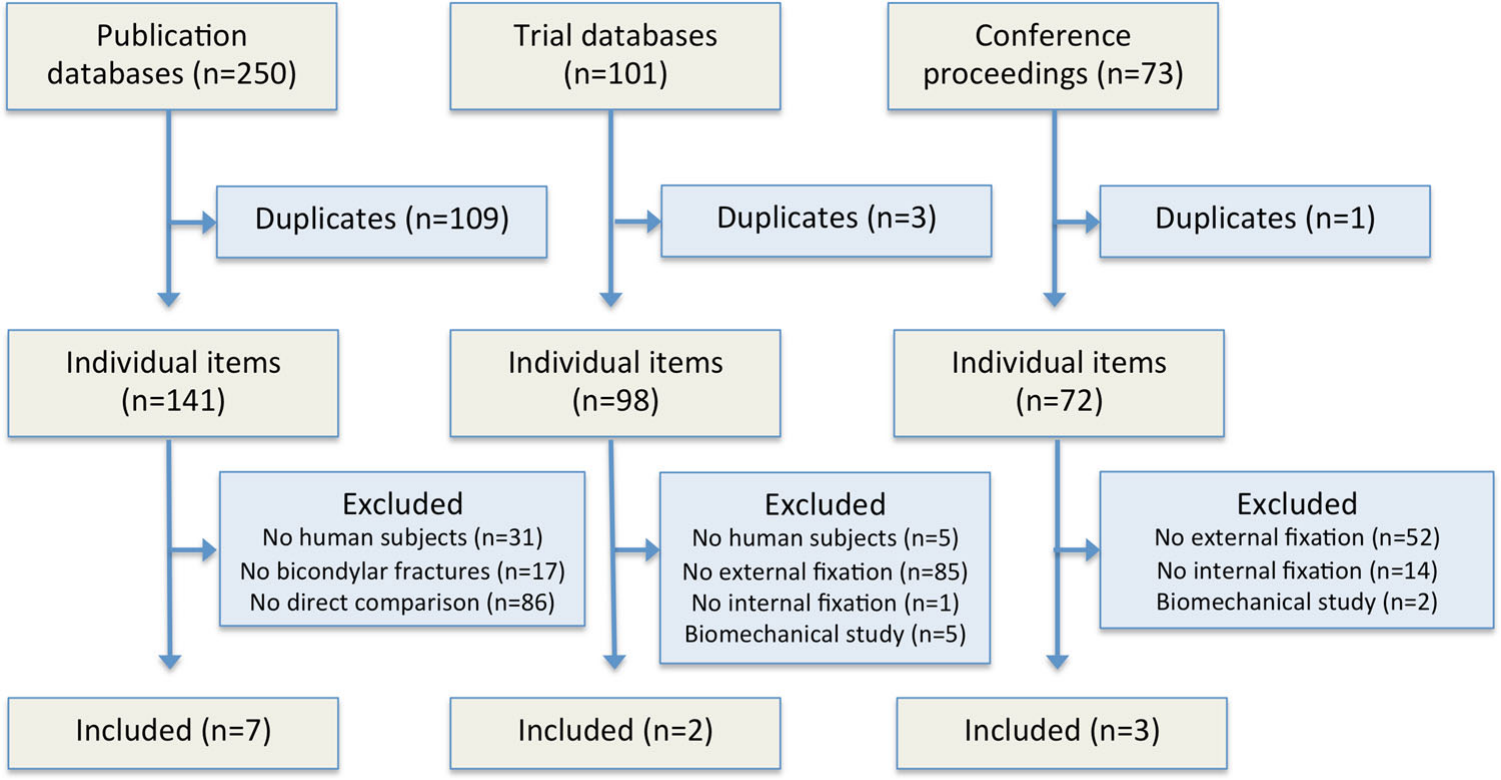
PRISMA flow diagram showing selection of studies for the systematic review

The initial search retrieved 311 individual items, of which ten satisfied the inclusion criteria (Fig. 1). These included seven full research papers [25–31], and three published conference abstracts [32–34], the characteristics of which are described in Table 1. Two registered trials were identified, both of which were represented by published studies retrieved during the search [28, 30]. Six items [26, 29, 30, 32–34] described three overlapping datasets and were analyzed in aggregate form as Boston [26, 29], Chertsey [33, 34], and COTS [30, 32].

**Table 1:** Key features of included studies

Name	Design	Patients (knees)		Interventions	Follow-up
		External fixation	ORIF		
Ahearn [14]	Retrospective	21	34	External fixation: Taylor Spatial Frame (TSF)^a^ ORIF: *Incision*: Unknown. *Fixation*: Lateral locking plate ± medial plate fixation	External fixation: mean 31 months (range 12–58 months) ORIF: mean 41 months (range 12–64 months)
Boston Mallik [29] Covall [26]	Retrospective	10 (10)	7 (7)	External fixation: Monticelli-Spinelli^b^ circular fixator ORIF: *Incision*: Unknown. *Fixation*: Bilateral buttress, semi-tubular plates, or cannulated screws	External fixation: mean 10 months (range 5–28 months) ORIF: mean 33 months (range 6–60 months)
Chertsey Guryel [34] Nawaz [33]	Retrospective	(79)	(45)	External fixation: Ilizarov circular frame ORIF: *Incision*: Unknown. *Fixation*: Unknown	Unknown
COTS McKee [30] Pirani [32]	Randomized controlled trial	42 (43)	40 (40)	External fixation: Closed/percutaneous/limited reduction, percutaneous lag screw, and Ilizarov circular frame ORIF: *Incision*: Single anterior or combined medial/lateral. *Fixation*: medial and lateral non-locking buttress plates ± iliac crest bone grafting	6, 12, and 24 months post-injury
Chan [25]	Retrospective	34 (35)	24 (24)	External fixation: Ilizarov circular frame (23/35, 65.7 %), Hoffman II^b^ with limited internal fixation (13/35, 37.1 %) ORIF: *Incision*: Unknown. *Fixation*: Buttress plate (21/24, 84 %), Less Invasive Stabilization System^c^ (4/24, 16 %)	3, 6, 12, and 24 months post-injury
Jansen [5]	Retrospective	2 (2)	20 (21)	External fixation: Synthes AO^c^ fixator or Ilizarov circular frame ORIF: *Incision:* Unknown. *Fixation*: LISS^c^ (19/20, 95.0 %) ± additional plates (7/20, 30.4 %) ± artificial bone substitute (7/20, 30.4 %)	Mean 67 months (range 36–109 months)
Krupp [28]	Retrospective	30 (30)	28 (28)	External fixation: Hoffman II Hybrid^b^ (16/28, 57.1 %) or circular (14/28, 50.0 %) frames and interval ORIF (locking plate or LISS^c^) ORIF: *Incision*: Unknown. *Fixation*: locking plate (8/28, 28.6 %), LISS^c^ (20/28, 71.4 %)	Mean unknown (range 6–53 months)

^a^Smith and Nephew Ltd, Brough, United Kingdom

^b^Stryker Corporation, Kalamazoo, MI

^c^DePuy Synthes Companies, West Chester, PA

There was one RCT and six retrospective studies reporting data on 419 fractures, of which 220 (52.5 %) were treated with external fixation.

### Study characteristics

The RCT [30, 32] was a large multi-centre trial in which patients with bicondylar tibial plateau fractures were randomized to either ORIF (with medial and lateral plates) or application of a circular fixator with percutaneous/limited open fracture reduction. The primary outcome measure was the Hospital for Special Surgery (HSS) knee score, which incorporates pain, function, range of motion, muscle strength, flexion contractures, and instability [35]. In total, 82 patients (83 fractures) were randomized, which was the number determined by an a priori power analysis designed to give an 80 % chance of detecting a 25 % mean difference in the primary outcome measure between the two groups.

The six retrospective studies [25–29, 31, 33, 34] accounted for 336 (80.2 %) of the published cases available for analysis. There was substantial heterogeneity in terms of the interventions used between the retrospective studies. Each reported on a range of external fixation and ORIF techniques using multiple devices. The former included Ilizarov circular frames, the Hoffman II (Stryker, Kalamazoo, MI), and the Synthes AO fixator (DePuy Synthes, West Chester, PA). ORIF techniques variously utilized locking plates, non-locking plates, and the Synthes Less Invasive Stabilization System (LISS) (DePuy Synthes, West Chester, PA). Some patients treated with ORIF also received iliac crest bone grafting or artificial bone substitute.

### Study quality

The RCT [30] was assessed to be at low risk of bias across most domains (Table 2), although there was no blinding of patients or personnel and the protocol was not published before recruitment commenced. For this reason, the study was judged to be at unclear risk of reporting bias. Financial support was received from Smith & Nephew Ltd (London, UK) and the Simon Fraser Orthopaedic Fund. Smith & Nephew sell a range of external fixation devices and it was not possible to determine whether the latter sponsor represented a commercial interest. There was no explicit statement as to the role of these funders in the study report.

**Table 2:** Risk of bias assessment of randomized studies

	Sequence generation	Allocation concealment	Blinding of participants, personnel and outcome assessors	Incomplete outcome data	Selective outcome reporting	Other sources of bias
COTS McKee [30] Pirani [32]	Low risk	Low risk	High risk	Low risk	Unclear risk	Low risk

Table 3 shows the risk of bias assessments for the six retrospective studies using the RoBANS tool [23]. Five were assessed to be at low risk of selection bias [25, 26, 28, 29, 31, 33, 34] and the remaining study was at unclear risk [27]. Low risk studies either declared that the series was consecutive or that it represented all cases treated over a given time period. No study explicitly reported blinding of outcome assessors and so all were assessed to be at unclear risk of detection bias. Similarly, the risk of reporting bias (selective outcome reporting) was unclear for all of the retrospective studies. Four studies were at high risk of attrition bias (incomplete outcome data) as a number of cases were lost to follow-up [25, 26, 28, 29, 31]. The remaining two were judged to be at low risk as outcome data was reported for almost all cases [27, 33, 34].

**Table 3:** Risk of bias assessment of non-randomized studies

	Selection of participants	Confounding variables	Intervention measurement	Blinding of outcome assessment	Incomplete outcome data	Selective outcome reporting
Ahearn [31]	Low risk	High risk	Low risk	Unclear risk	High risk	Unclear risk
Boston Mallik [29] Covall [26]	Low risk	High risk	Low risk	Unclear risk	High risk	Unclear risk
Chertsey Guryel [34] Nawaz [33]	Low risk	High risk	Low risk	Unclear risk	Low risk	Unclear risk
Chan [25]	Low risk	High risk	Low risk	Unclear risk	High risk	Unclear risk
Jansen [5]	Unclear risk	High risk	Low risk	Unclear risk	Low risk	Unclear risk
Krupp [28]	Low risk	High risk	Low risk	Unclear risk	High risk	Unclear risk

The retrospective studies were all judged to be at high risk of confounding variables. Four of the retrospective studies addressed known confounders by reporting the patient characteristics of each group. Such reporting was, however, limited and variable [25, 26, 28, 29, 33, 34]. Only Chan et al. described a significant difference between the two groups in that alcohol dependency was over-represented in the external fixation group (4 % vs 20 %). Jansen et al. described demographic characteristics for their whole series but not by treatment modality [27]. Due to their retrospective nature, additional confounders (either unreported or unidentified) are likely to exist and conclusions from these studies should therefore be treated with caution.

### Radiographic outcomes

Two studies (142 fractures) assessed fracture reduction radiologically [25, 30]. In both studies, a single assessor graded post-operative radiographs. Chan et al. additionally scored radiographs using Rasmussen’s system, which is based on joint depression, condylar widening, and varus/valgus angulation [10]. Although designed specifically for fractures around the knee, there is little published evidence assessing its reliability and validity [36]. These studies reported no statistically significant differences in terms of articular displacement, diaphyseal-metaphyseal angulation/translation, condylar widening, or Rasmussen’s score.

Only Krupp et al. [28] reported time to radiographic union which was comparable between the two groups: 6 (range 3–14) months in the ORIF group and 7 (range 3–15) months in those managed with external fixation.

**Fig. 2:**
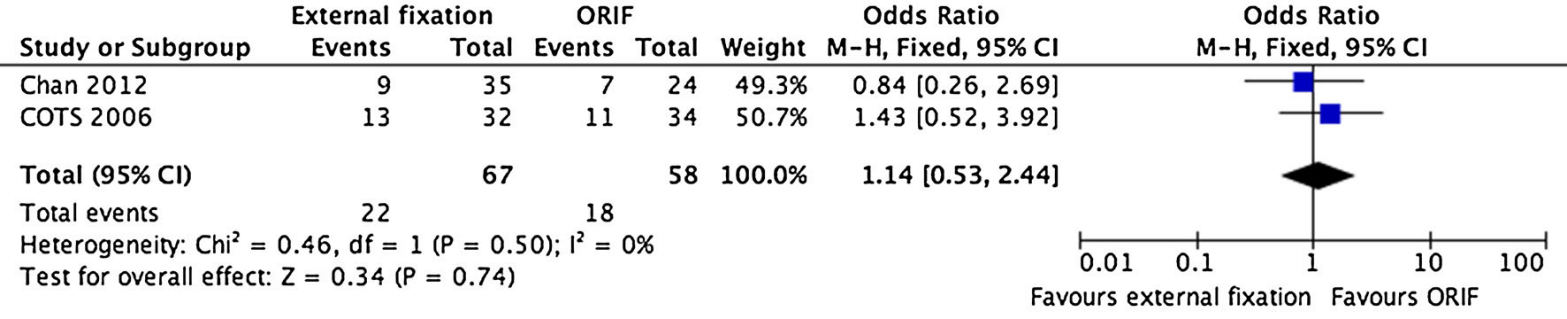
A forest plot showing pooled data from studies reporting radiographic evidence of OA at 24 months post-injury

Three studies (165 fractures) assessed follow-up radiographs for evidence of OA [25, 27, 30]. The COTS and Chan studies both used radiographs taken after the same standardized follow-up period, i.e., 24 months post-operatively. However, they relied on subjective assessment by a single unblinded assessor. Jansen et al. increased the reliability of their results by using an established radiographic interpretation tool: the Kellgren-Lawrence score [37]. Unfortunately they reported onset of OA for their series as a whole without distinguishing between the two treatment groups. Their follow-up period also ranged from 36 to 109 months, making it difficult to directly compare patients [27]. Pooled results from the remaining two studies (Fig. 2) found radiographic evidence of OA in 22 (32.8 %) of external fixation and 18 (31.0 %) of ORIF cases (OR 1.14, 95 % CI 0.53–2.44, *P* = 0.740) at 24 months post-injury.

### Functional outcomes

Three studies reported functional outcomes [30–34]. Although Krupp et al. reported better range of movement in the ORIF group, they provided no indication of statistical significance. In general, there were few significant differences between the groups on any functional outcome. The COTS primary outcome measure (HSS) [35] trended towards higher HSS in the external fixation group (mean difference in HSS 11.00, 95 % CI 2.03–19.97, *P* = 0.06), which might have reached significance with a greater sample size. However, any genuine difference did not persist at 12 (mean difference 5.00, 95 % CI −2.59 to 12.59, *P* = 0.406) and 24 months (mean difference 7.00, 95 % CI −1.45 to 15.45, *P* = 0.307) Similarly, the external fixation patients were more likely to have returned to pre-injury activities at 6 months (*P* = 0.030) but not at later follow-up assessments.

Jansen et al. reported outcomes for their whole series using the Lysholm score [38] and Knee Injury and Osteoarthritis Outcome Score (KOOS) [39] but did not distinguish between patients in the two treatment groups.

### Subsequent knee arthroplasty

**Fig. 3:**
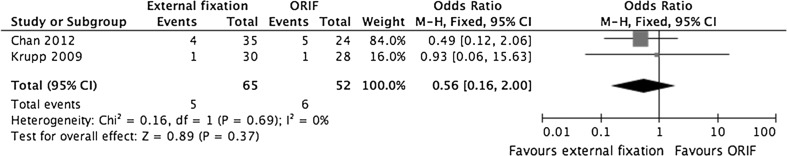
A forest plot showing pooled data from studies reporting need for subsequent total knee replacement

Two studies (117 fractures) reported on subsequent need for ipsilateral total knee arthroplasty (TKA) [25, 28]. Figure 3 shows that the pooled rates of TKA in the external fixation and ORIF groups were 7.7 and 11.5 % (OR 0.56, 95 % CI 0.16–2.00, *P* = 0.69). Chan et al. followed up patients at 24 months, although it is uncertain whether TKAs occurring subsequently were included. For example, they reported cases presenting before March 2005 but published their paper in 2012. The authors do not state whether TKAs were included if performed between 2005 and 2012. The cases reported by Krupp et al. had variable follow-up lengths that ranged from 6 to 53 months. In any event, it is likely that an unknown proportion of patients developed end-stage post-traumatic OA requiring TKA outside these follow-up periods.

### Complications

**Fig. 4:**
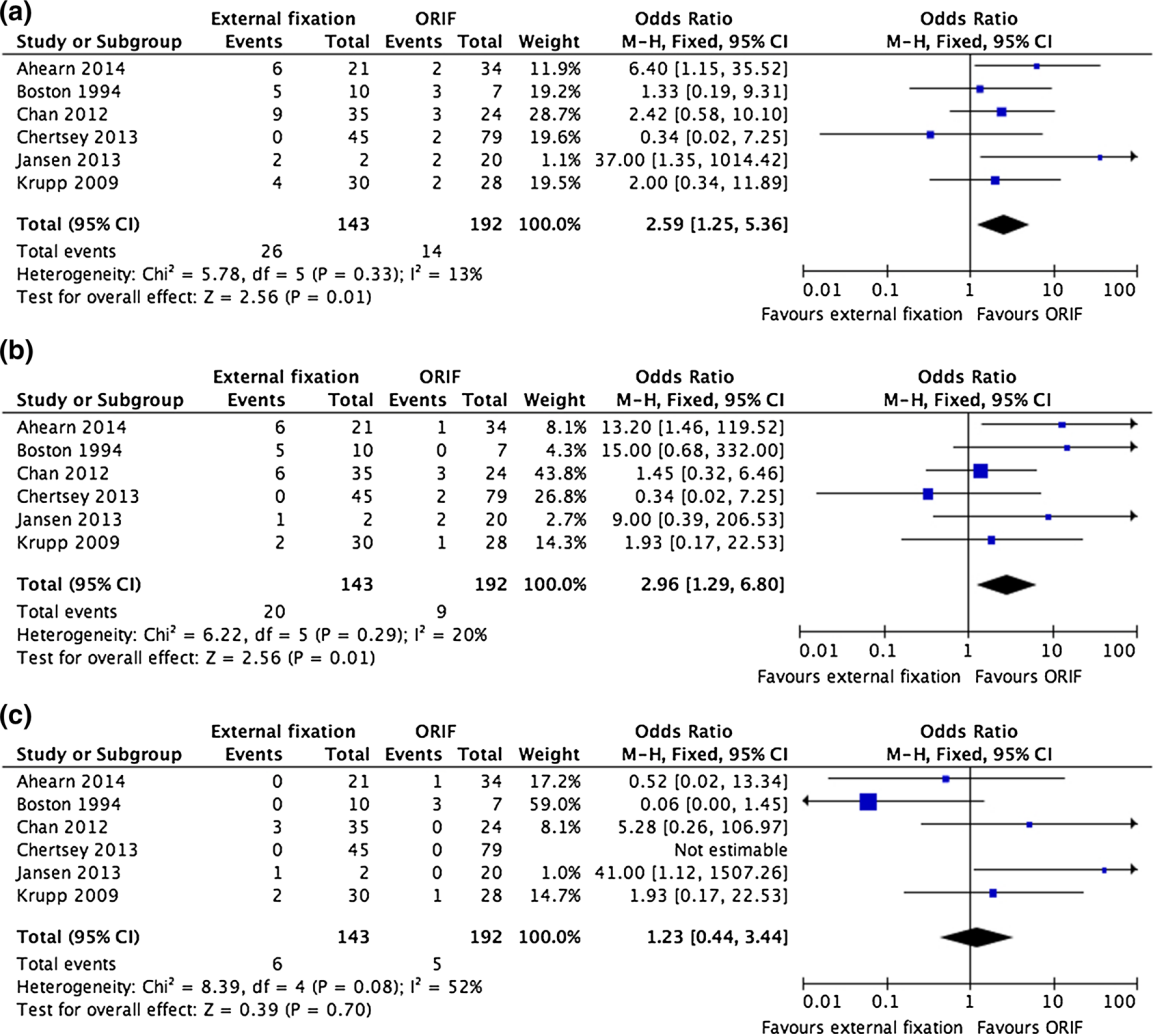
**a** A forest plot showing pooled results of studies reporting all post-operative infections, **b** a forest plot showing pooled results of studies reporting superficial post-operative infections, and **c** a forest plot showing pooled results of studies reporting deep post-operative infections

All six retrospective studies (336 fractures) described rates of superficial and deep infection [25–29, 31, 34]. The rates of superficial infection in the external fixation and ORIF groups, respectively, were 14.0 vs 4.7 % (OR 1.93, 95 % CI 0.17–22.53, *P* = 0.01). The rates of deep infection were 4.2 and 2.6 % (OR 1.23, 95 % CI 0.44–3.44, *P* = 0.700), respectively. Pooled results for any infection (deep or superficial) found that patients treated with external fixation had greater odds of this outcome (OR 2.59, 95 % CI 1.25–5.36, *P* = 0.01). The forest plots for these infections are shown in Fig. 4.

**Fig. 5:**
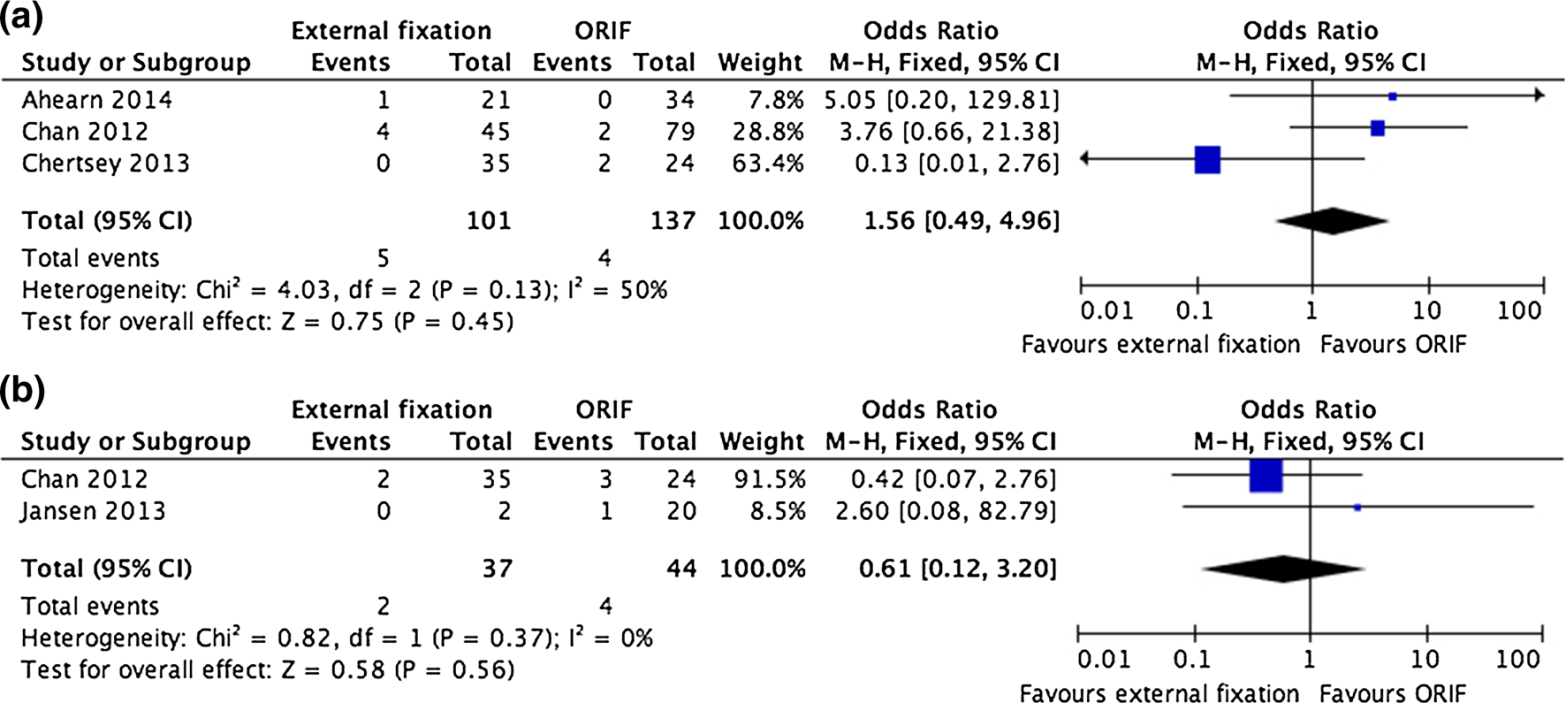
**a** A forest plot showing pooled data from studies reporting on rates of venous thromboembolism and **b** a forest plot showing pooled data from studies reporting on rates of compartment syndrome

Three studies (238 fractures) described rates of venous thromboembolism (VTE) [25, 31, 34]. There were nine cases of deep vein thrombosis (3.8 %), with no statistically significant differences between the groups (OR 1.56, 95 % CI 0.49–4.96, *P* = 0.45), and no reported pulmonary emboli. As neither study described screening for VTE, these cases presumably presented symptomatically. Compartment syndrome was reported as a complication by two studies (81 fractures) [25, 27]. It featured in 5.4 % of external fixation cases and 9.1 % of those undergoing ORIF (OR 0.61, 95 % CI 0.12–3.20, *P* = 0.56). Forest plots for VTE and compartment syndrome are shown in Fig. 5.

### Re-operation

**Fig. 6:**
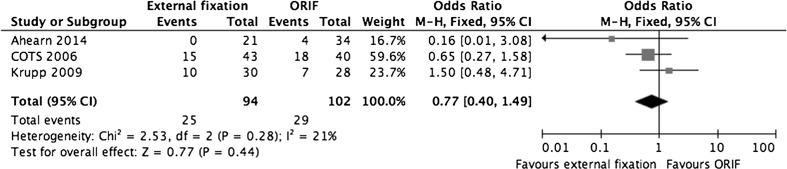
A forest plot showing pooled data from studies reporting need for subsequent re-operation

Three studies (196 fractures) described rates of re-operation, as shown in Fig. 6 [28, 30, 31]. In the pooled external fixation group, 25 cases (26.6 %) required an additional 40 operations whereas, in the ORIF group, 29 (28.4 %) required 72 operative interventions. The pooled re-operation rate was not statistically significant (OR 0.77, 95 % CI 0.40–1.49, *P* = 0.44). However, no study took planned procedures (such as frame removal) into account during their analyses. In the COTS trial, 27 frames (65.9 %) were removed in the operating theatre under general anaesthetic or sedation.

Substantial re-operations (e.g., knee arthrodesis) in the ORIF group were described in the Boston series, although these papers did not describe re-operations systematically. The COTS report observed that re-operations following ORIF were more substantial (e.g., above knee amputation, osteotomy) than in the external fixation group (e.g., pin-track debridement), although there was no attempt to quantify this observation.

## Discussion

Although ORIF is often successful in restoring articular congruity, it may further compromise the soft tissue envelope. Many case series have highlighted the dangers of wound breakdown and deep infection following ORIF of bicondylar tibial plateau fractures [8, 29, 40]. These problems have persisted, even in modern studies utilizing techniques such as delayed surgery and minimal soft tissue dissection. For example, Baeri et al. reported deep infections in seven (8.4 %) of 83 patients treated with ORIF, each of whom required a mean 3.3 additional operations as a consequence [41].

External fixation devices preserve soft tissues and an emerging body of evidence suggests they can achieve lower rates of deep infection [42–44]. Although external fixation might risk sacrificing the quality of fracture reduction, it is uncertain whether this ultimately affects functional outcome [9–12].

Few studies have directly compared external fixation and ORIF for treatment of bicondylar tibial plateau fractures. This systematic review identified seven such studies, most of which were poor-quality retrospective case series, although there was one RCT. There was substantial heterogeneity of study populations and reported outcomes. In addition, the retrospective studies, which accounted for the majority of cases available for analysis (80.2 %), were at high risk of bias caused by confounder variables. Pooled data from these studies suggests that patients managed with external fixation are at greater risk of superficial infection, although other complications (including deep infection) were comparable between the groups. However, patients undergoing external fixation may return to pre-injury activities faster than those treated with ORIF. The seven studies identified no other statistically significant differences across a range of outcomes between ORIF and external fixation.

One important limitation of all existing studies is the relatively short follow-up duration. Post-traumatic OA is an important long-term complication of intra-articular fractures through this weight-bearing joint. However, it is difficult to rely on reported rates of secondary OA and need for subsequent TKA in these studies, given the small numbers involved, short follow-up durations, and inconsistent reporting. Similarly, review of follow-up radiographs for early evidence of OA relied on subjective interpretation by non-blinded assessors. Although there are few short-term functional differences between those undergoing ORIF and external fixation, the long-term impact on knee OA remains unknown. Importantly, the three studies assessing quality of articular surface restoration found no difference between the two groups [25, 30, 31].

There is additional uncertainty surrounding the complication profile of the two procedures. Although the proportion of patients requiring re-operation appeared to favour external fixation, this was not statistically significant. However, the analysis did not include planned procedures, including the need for frame removal under sedation and/or anaesthesia. It was also suggested that re-operations following ORIF may be of greater importance than those following external fixation [30]. Importantly, infection complicated a greater proportion of cases managed with an external fixator than with ORIF (OR 2.59, 95 % CI 0.49–4.96). This suggests that the soft tissue complications of external fixation could be even greater than ORIF in this setting.

The existing evidence suggests that neither ORIF nor external fixation is clearly superior in the management of bicondylar tibial plateau fractures. Importantly, external fixation does not offer any clear advantage over ORIF in terms of avoiding soft tissue complications. Although clinicians should be mindful of subtly different complication profiles and the possible need to remove external fixators in theatre, both external fixation and ORIF are acceptable strategies for managing these injuries.
